# Somatosensory Profile of Central Post Stroke Pain of Thalamic Origin: Findings of a Quantitative Sensory Testing Study

**DOI:** 10.1002/ejp.70104

**Published:** 2025-08-15

**Authors:** Kristel Berati, Lukas Enz, Priska Zuber, Katarina Alexandra Ebner, Shaumiya Sellathurai, Kean Schoenholzer, Federico Burguet Villena, Laura Gaetano, Ludwig Kappos, Stefano Magon, Till Sprenger, Athina Papadopoulou

**Affiliations:** ^1^ Clinic of Neurology University and University Hospital of Basel Basel Switzerland; ^2^ Department of Clinical Research University Hospital Basel and University of Basel Basel Switzerland; ^3^ Health, Medical and Neuropsychology Institute of Psychology, Leiden University Leiden the Netherlands; ^4^ Novartis Basel Switzerland; ^5^ Research Center for Clinical Neuroimmunology and Neuroscience Basel (RC2NB) University Hospital Basel and University of Basel Basel Switzerland; ^6^ Pharma Research and Early Development, Neuroscience and Rare Diseases Roche Innovation Center Basel F. Hoffmann‐La Roche Ltd. Basel Switzerland; ^7^ University Hospital Zürich Zürich Switzerland

**Keywords:** CPSP, mechanical pain sensitivity, phenotyping, QST, thalamic pain, thalamic stroke

## Abstract

**Introduction:**

Central post stroke pain (CPSP) is attributed to vascular lesions of the central somatosensory system, including the thalamus.

**Objective:**

A better characterisation of clinical findings in patients with CPSP after thalamic stroke can facilitate research and treatment of this refractory pain syndrome. We aimed to quantify somatosensory abnormalities in CPSP patients after thalamic stroke.

**Methods:**

Sixteen patients with CPSP after thalamic stroke, 14 patients with a history of thalamic stroke without any pain (stroke control patients, SCP) and 12 healthy controls (HC) underwent detailed clinical examination, standardised quantitative sensory testing (QST) and a pain questionnaire. QST results were compared to age and sex adjusted reference data to obtain *z*‐scores. Group comparisons were performed with one‐way analysis of variance.

**Results:**

Temperature perception did not differ in CPSP patients, apart from thermal sensory limen (higher in CPSP vs. HC but no difference vs. SCP). Patients with CPSP showed higher mechanical detection thresholds compared to SCP (Δ = 1.26, *p* = 0.017, no difference vs. HC) and they were more sensitive to mechanical pain than SCP (lower mechanical pain thresholds vs. SCP: Δ = −1.32, *p* = 0.014, no difference vs. HC).

**Conclusion:**

Our results indicate somatosensory abnormalities in patients with CPSP after thalamic stroke, associated with the perception of mechanical stimuli. A short somatosensory screening including mechanical perception may contribute to an accurate diagnosis of this debilitating condition.

**Significance Statement:**

Thalamic CPSP is very rare, thus our data contribute to the clinical and sensory phenotyping of patients suffering from this debilitating condition. We did not find abnormalities in *thermal* measures, but only *mechanical* thresholds in our cohort, suggesting that not only changes in temperature perception are necessary for the development of pain after thalamic stroke. Our findings suggest that a short QST protocol including *mechanical* testing using von Frey filaments and pin‐prick‐stimulators may be useful in the diagnosis of thalamic CPSP.

## 1 Introduction

Central post stroke pain (CPSP) is a neuropathic chronic pain syndrome, attributed to a vascular lesion within the central nervous system (CNS) (Bowsher 1996; Klit et al. 2009; Loeser and Treede 2008; Treede et al. 2008). Although the lesion can be located anywhere along the central somatosensory system, CPSP was first described in patients with thalamic lesions by Dejerine and Roussy as “among the most spectacular, distressing, and intractable of pain syndromes” (Déjerine and Roussy 1906; Henry et al. 2008).

The prevalence of CPSP is reported between 1% and 12% among all strokes along the central somatosensory system (Boivie et al. 1989; Kong et al. 2004), while it may be much higher in patients with pure sensory stroke (Asseyer et al. 2025; Garcia‐Larrea et al. 2010; Harno et al. 2014; Paciaroni and Bogousslavsky 1998).

Taking into account the increasing prevalence of stroke in the aging population (Truelsen et al. 2006), the incidence of CPSP is expected to increase over time. In thalamic strokes, the CPSP prevalence was estimated from 25% (Bogousslavsky et al. 1988; Bowsher et al. 1998) to 33% (Andersen et al. 1995; Leijon et al. 1989; Widar et al. 2002). The exact location of the stroke lesion in the ventral posterior thalamus is associated with a higher risk of developing CPSP (Bowsher et al. 1998; Krause et al. 2012; Sprenger et al. 2012; Vartiainen et al. 2016). Additionally, a recent study (Delboni Lemos et al. 2022) reported that thalamocortical white matter changes also play a significant role in the development of CPSP. However, the mechanisms involved in the pathogenesis of pain are not fully understood.

Clinical research on CPSP treatment is limited. The only study on prophylactic treatment of CPSP to our knowledge is a prospective placebo‐controlled trial with amitriptyline (Lampl et al. 2002), which was, however, likely underpowered (total *n* = 39, only 17%–21% of patients with stroke developed CPSP). The assessment of a specific somatosensory profile associated with CPSP of thalamic origin would help to detect subjects at higher risk to develop CPSP and thus facilitate prospective studies. Moreover, the exact characterisation of sensory symptoms linked to thalamic CPSP is relevant from a clinical point of view to assure an early, correct diagnosis and improve prognosis.

Prior studies using quantitative sensory testing (QST), a standardised method that is feasible both in clinical studies and routine, reported differences between patients with CPSP and stroke patients without pain (Asseyer et al. 2025; Barbosa et al. 2022; Krause, Asseyer, Geisler, et al. 2016). They however included heterogeneous groups of patients with both thalamic and extra‐thalamic strokes. In our study, we aimed at characterising somatosensory abnormalities associated specifically with CPSP of *thalamic* origin using QST. Moreover, we aimed at identifying the QST thresholds that are most accurate to differentiate between stroke patients with and without CPSP.

Our secondary, more explorative objective was to investigate potential patterns regarding the distribution of pain and associated sensory symptoms (positive and negative) in thalamic CPSP patients.

## 2 Methods

### 2.1 Study Participants: Criteria, Definitions and Neurological Assessment

Overall, 30 patients with a history of thalamic stroke (16 with CPSP and 14 without pain = “stroke control patients, SCP”) and 12 healthy controls (HC) were included in the study (Table 1). Eligible patients were identified from the database of the Clinic of Neurology, University Hospital Basel and were actively invited to participate in this study. HC were enrolled via advertisement in public notice boards.

**TABLE 1:** Baseline characteristics of all study participants.

Characteristics	Thalamic CPSP (*n* = 16)	Stroke control patients (*n* = 14)	Healthy controls (*n* = 12)	Group comparison
Age: mean ± SD, y	62.6 ± 17.7	65.0 ± 15.9	69.3 ± 10.6	One‐way ANOVA *p* = 0.524
Sex: F/M (F %)	8/8 (50%)	7/7 (50%)	4/8 (33%)	Fisher's Exact Test, *p* = 0.681
Time since stroke: median (Min–Max), months	25 (1–249)^a^	63 (23–98)	—	Wilcoxon–Mann–Whitney test (*p* = 0.044)
Stroke side R/L (R %)	8/8 (50%)	9/5 (64%)	—	Fisher's Exact Test, *p* = 0.484
Initial NIHSS score: median (minimum‐maximum)	4 (1–11)	2 (1–16)	—	Wilcoxon–Mann–Whitney test (*p* = 0.450)
Hypoesthesia at time of stroke: *n* (%)	14 (88%)^b^	9 (65%)	—	Fisher's Exact Test, *p* = 0.204
Hypoesthesia at study visit: *n* (%)	11 (69%)	7 (50%)	—	Fisher's Exact Test, *p* = 0.457
Stroke type: Ischemic/Hemorrhagic (Ischemic %)	14/2 (88%)	10/4 (71%)	—	Fisher's Exact Test, *p* = 0.378
Ischemic stroke Aetiology: *N* (%)			—	
Small‐vessel disease (lacunar)	8 (50%)	4 (29%)		—
Cardioembolic	3 (19%)	5 (36%)		—
Large‐artery atherosclerotic	1 (6%)	1 (7%)		—
Vertebral dissection	1 (6%)	0		—
Undetermined	1 (6%)	0		—
Hemorrhagic stroke Aetiology: *N* (%)			—	
Cavernoma	1 (6%)	0		—
Arteriovenous malformation	1 (6%)	1 (7%)		—
Hypertension	0	1 (7%)		—
Cerebral amyloid angiopathy	0	1 (7%)		—
Unknown	0	1 (7%)		—

*Note:* The NIHSS score refers to the time of acute stroke diagnosis.

Abbreviations: CPSP, central post‐stroke pain (here: of thalamic origin); F, female; L, left; M, male; NIHSS, National Institutes of Health Stroke Scale; R, right; SD, standard deviation; y, years.

^a^ A single patient had the stroke (and pain onset) 21 years before the study baseline.

^b^ Two patients had no hypoesthesia at the time of stroke, but initially had par‐, dys‐, hyperesthesia and allodynia.

Inclusion criteria of the three groups were: For patients with CPSP: (i) age: ≥ 18 years old, (ii) history of unequivocal thalamic stroke (both infarction or bleeding), (iii) diagnosis of definite neuropathic central‐post stroke pain, according to the criteria proposed by Klit et al. (2009), which were applied at study baseline, based on detailed history and neurological examination, (iv) the most significant pain syndrome (i.e., the most intense and frequent one) should fulfil the criteria for neuropathic pain. No nociceptive, peripheral neuropathic, or other chronic pain condition or disorder potentially confounding the aetiology of the most significant pain (e.g., spasticity and contractures) should be present.

For the SCP: (i) age: ≥ 18 years old, (ii) history of unequivocal unilateral thalamic stroke (infarction or bleeding) at least two years ago (to minimise the possibility that these patients might still develop CPSP), (iii) no nociceptive, peripheral, or central neuropathic pain any time since stroke and no chronic pain condition in general.

For HC: (i) age ≥ 18 years old; (ii) no history of stroke in the thalamus or any other stroke or other known brain injury; (iii) no chronic pain condition.

The exclusion criteria for all three groups were: (i) history of severe neurological or psychiatric disease (other than stroke) (ii) unable to perform MRI (e.g., pacemaker, claustrophobia, pregnancy).

All patients underwent a detailed comprehensive assessment at study baseline, including neurological history and a neurological examination, according to standard clinical practice, by an experienced neurologist (AP). This included light touch by using cotton swab, superficial pain by using the sharp part of a toothpick, joint position by blind movement of finger and toe and vibration sense by using a turning fork 128 Hz.

Patients in the CPSP group already had a diagnosis of “thalamic pain syndrome” from their treating neurologists. To ensure that they all indeed experienced neuropathic CPSP (and not other stroke‐related pain, e.g., due to spasticity‐ or muscular‐pain), the diagnosis was confirmed by two certified neurologists, using the criteria proposed by Klit et al. (2009). Accordingly, patients in the SCP also had a detailed interview and neurological examination to exclude the possibility of CPSP (or other pain).

Moreover, most subjects (10/16 of the CPSP patients and all SCP) underwent a brain MRI within 15 days of the QST examination, to confirm the symptomatic thalamic lesion. No new thalamic lesions were detected. The six CPSP patients that did not have an MRI at the time of QST had a previous MRI at the time of stroke, with in all cases symptomatic unilateral thalamic lesion.

Clinical sensory findings were separately recorded for the various body parts and divided into negative (hypoesthesia) and positive (including par−/dysesthesia, hyperalgesia and allodynia).

For this analysis, the body was divided into the following segments: face, upper arm, forearm, hand, trunk, upper leg, lower leg, and foot.

One screened patient with bilateral thalamic lesions and bilateral peripheral neuropathic pain in the legs (probably due to small fibre neuropathy) and one with unilateral thalamic stroke but only dysesthesias, not fulfilling the definition of CPSP (Klit et al. 2009), were excluded from the analysis. One screened stroke‐control patient with migraine was excluded from the analysis.

The study was approved by the local ethics committee (Ethikkommission‐Nordwest‐ Zentralschweiz; EKNZ‐number: 2014–308) and conducted in accordance with the declaration of Helsinki. All participants gave written informed consent before inclusion in the study.

### 2.2 Pain Questionnaire

The CPSP group completed a standardised, widely accepted, pre‐validated questionnaire in the German language and comprehensive pain questionnaire from the German Pain Association to further characterise the neuropathic pain syndrome (Deutscher Schmerzfragebogen), DGSS, copyright: https://www.schmerzgesellschaft.de/ (Petzke et al. 2020). The DGSS consists of 15 multiple‐choice questions covering a detailed description of the patients' predominant pain, including localisation, character, frequency, duration, intensity, variations in time as well as its accompanying symptoms and impact on daily life. Additionally, it features two open‐ended questions allowing patients to describe their pain in their own words.

### 2.3 Quantitative Sensory Testing

A standardised QST‐battery was performed in all three groups, according to the protocol described by the German Research Network for Neuropathic pain (DFNS) (Rolke et al. 2006). The standardised QST‐protocol consisted of the following tests:

- Temperature testing (https://www.medoc‐web.com/pathway) for assessment of heat‐ and cold detection‐ and pain thresholds, as well as thermal sensory limen procedure (TSL). The thermal sensory limen procedure was performed using a thermal sensory testing device (Medoc, Israel). The baseline temperature was 32°C. The device was successively heated or cooled at a rate of 1°C per second. To detect the thermal sensory limen, a series of alternating cold and warm stimuli were applied to the tested area; this was repeated 6 times. The patients were instructed to indicate each change in temperature perception as cold, warm, or heat/burning pain. Thus, during this procedure, paradoxical heat sensation (PHS) was assessed as a sensation of warmth, heat, or burning pain on a cooling stimulus.
- Mechanical detection threshold (https://www.mrc‐systems.de/de/produkte/pinprick) (von Frey filaments)
- Mechanical pain threshold and mechanical pain sensitivity (https://www.mrc‐systems.de/de/produkte/pinprick) (PinPricks)
- Dynamic mechanical allodynia (to light touch, e.g., by cotton wisp, brush etc.)
- Wind up ratio, as correlate for temporal pain summation (https://www.mrc‐systems.de/de/produkte/pinprick) (PinPricks)
- Vibration detection thresholds (tuning fork, 64 Hz, 8/8 scale)
- Pressure pain threshold (https://www.wagnerinstruments.com/products/Pain‐Test‐Algometers/fpx).

All tests were performed by two experienced raters who previously underwent specialised training and certification. Standardised instructions in the German language were given. QST was performed on the dorsal hand, according to the published protocol (Rolke et al. 2006). We chose the hand (instead of face/foot), since it is frequently affected by thalamic pain and less frequently affected by other peripheral neurological abnormalities than the foot. For both stroke groups, all tests were applied on both hands, in random order to avoid a systematic bias. In the HC group, QST was performed only on the left hand.

### 2.4 Statistical Analysis

All statistical analyses were performed in R version 4.2.2 (R Development Core Team 2010). Since our study was exploratory, we did not perform a sample size calculation. For the statistical analysis, we followed previously published recommendations (Magerl et al. 2010). Thus, the original data for cold−/warm‐detection (CDT, WDT), thermal sensory limen (TSL), pressure−/mechanical pain thresholds (PPT, MPT), mechanical pain sensitivity (MPS), wind‐up ratio (WUR), mechanical detection threshold (MDT) and dynamic mechanical allodynia (DMA) were log‐transformed with base 10. In addition, we visualised the log‐transformed data using QQ‐plots, which did not provide evidence for deviation from normal distribution. We used published reference data to calculate z‐scores for all QST parameters per patient by subtracting the age and sex specific mean per patient and dividing by the age and sex specific standard deviation (Magerl et al. 2010). All analyses were then performed with the z‐scores. To not introduce any selection bias, no data points were excluded from the analysis. To compare the groups, we calculated type III one‐way analysis of variance (ANOVA) and if the F‐test revealed a *p*‐value smaller than 0.05, a Tukey post hoc analysis was performed. Additionally, only for CPSP and SCP, an ANCOVA adjusting for time since stroke was calculated as a sensitivity analysis. For all other statistical tests, Fisher's exact test, paired t‐tests (within‐subject comparison) or the Wilcoxon–Mann–Whitney test were used as indicated. A *p*‐value or adjusted *p*‐value (Tukey‐post hoc) smaller than 0.05 was considered statistically significant. For the main group comparisons (ANOVA models), we used the false detection rate (FDR) to correct for multiple testing. In Table 2, we show both corrected and uncorrected *p*‐values. However, since our study was exploratory and of limited sample size, we report the results and discuss our findings based on the uncorrected *p*‐values. We also performed sensitivity analysis by (i) excluding five patients (CPSP 02, 03, 06, 14, 15) with additional ipsilesional pain (Table 3) and (ii) excluding one patient with an additional brain lesion (older stroke in the left frontoinsular cortex, CPSP 11).

**TABLE 2:** Raw values and group comparisons for all QST parameters.

	QST parameter	CPSP mean (±SD)	SCP mean (±SD)	HC mean (±SD)	ANOVA				ANCOVA adjusted for time since stroke
					Group‐comparison (*F*‐test)	CPSP vs. SCP	CPSP vs. HC	SCP vs. HC	CPSP – SCP
Thermal testing	Cold detection threshold [°C]	3.7 (±6.8)	3.1 (±2.4)	1.7 (±1.2)	*p* = 0.128	—	—	—	—
	Warm detection threshold [°C]	5.5 (±4.6)	5 (±3.5)	5 (±5.1)	*p* = 0.395	—	—	—	—
	Thermal sensory limen [°C]	10.7 (±6.7)	8.3 (±5.5)	5.6 (±4.8)	***p* = 0.003** (FDR: *p* = 0.038)	Δ = 0.38 (−0.43 to 1.18), *p* = 0.497	Δ **= 1.28 (0.42 to 2.14), *p* = 0.002**	Δ **= 0.91 (0.02 to 1.79), *p* = 0.044**	—
	Cold pain threshold [°C]	16 (±10.2)	13.4 (±9.7)	13.6 (±11.5)	*p* = 0.854	—	—	—	—
	Heat pain threshold [°C]	44.3 (±4.8)	42.8 (±4.5)	43 (±5.7)	*p* = 0.435	—	—	—	—
Mechanical testing	Mechanical detection threshold [mN]	9.9 (±7.1)	5.2 (±6.5)	3.8 (±3.1)	***p* = 0.014** (FDR: *p* = 0.058)	Δ **= 1.26 (0.19 to 2.32), *p* = 0.017**	Δ = 1.05 (−0.09 to 2.19), *p* = 0.077	Δ = −0.21 (−1.38 to 0.96), *p* = 0.902	Δ **= 1.32 (0.43 to 2.21), *p* = 0.007**
	Mechanical pain threshold [mN]	41.8 (±57.3)	84.7 (±64.2)	48.9 (±65.1)	***p* = 0.013** (FDR: *p* = 0.058)	Δ **= −1.32 (−2.4 to −0.23), *p* = 0.014**	Δ = −0.17 (−1.33 to 0.99), *p* = 0.93	Δ = 1.14 (−0.05 to 2.34), *p* = 0.063	Δ = (**−2.23 to −0.41), *p* = 0.008**
	Mechanical pain sensitivity [mN]	3.3 (±3.8)	1.8 (±1.9)	2.6 (±3.8)	*p* = 0.665	—	—	—	—
	Dynamic mechanical allodynia [no unit]	1.4 (±3.3)	0.7 (±2.2)	0.2 (±0.1)	*p* = 0.56	—	—	—	—
	Wind‐up ratio [no unit]	2.7 (±1.8)	2.1 (±1.3)	1.9 (±1.3)	*p* = 0.415	—	—	—	—
	Vibration detection threshold [no unit]	6.6 (±2)	6.8 (±0.8)	7.5 (±0.4)	*p* = 0.177	—	—	—	—
	Pressure pain threshold, [kPa]	315.3 (±51.1)	281 (±60.9)	337.2 (±75.1)	*p* = 0.18	—	—	—	—

*Note:* The mean and standard deviation values of the QST parameters for all groups are shown in original scale, but the group comparisons were performed using *z*‐scores, adjusted for age and sex, based on normative values. Note also that the pair‐wise group comparisons with Tukey post hoc test were performed only if the *F*‐test of ANOVA had a *p*‐value < 0.05 (thus only for: thermal sensory limen, mechanical detection threshold and mechanical pain threshold). The false detection rate (FDR) multiple test correction was applied over all *p*‐values from the ANOVA group comparison (left‐most ANOVA column); the results are only reported for raw *p*‐values below 0.05. The sensitivity analysis with additional adjustment for time since stroke (only between thalamic CPSP and SCP) is shown in the last right column. *p*‐values (< 0.05) are marked in bold. Please note that the results refer to the contralesional side and the left side in healthy individuals.

Abbreviations: ∆, pairwise difference of the means; °C, degree Celsius; CPSP, central post‐stroke pain (here: of thalamic origin); FDR, false discovery rate; HC, healthy controls; kPa, kilopascal; mN, millinewton; QST, quantitative sensory testing; SCP, stroke control patients.

**TABLE 3:** Group comparisons for all QST parameters: Sensitivity analysis.

	QST parameter	ANOVA			
		Group‐comparison (*F*‐test)	CPSP vs. SCP	CPSP vs. HC	SCP vs. HC
Thermal Testing	Cold detection threshold [°C]	*p* = 0.074	—	—	—
	Warm detection threshold [°C]	*p* = 0.241	—	—	—
	Thermal sensory limen [°C]	***p* = 0.001** (FDR: *p* = 0.011)	Δ = 0.61 (−0.24 to 1.46), *p* = 0.194	Δ **= 1.52 (0.62 to 2.42), *p* = 0.001**	Δ **= 0.91 (0.06 to 1.76), *p* = 0.035**
	Cold pain threshold [°C]	*p* = 0.895	—	—	—
	Heat pain threshold [°C]	*p* = 0.538	—	—	—
Mechanical Testing	Mechanical detection threshold [mN]	***p* = 0.010** (FDR: *p* = 0.062)	Δ **= 1.43 (0.29 to 2.56), *p* = 0.011**	Δ **= 1.22 (0.02 to 2.42), *p* = 0.046**	Δ = −0.21 (−1.34 to 0.93), *p* = 0.894
	Mechanical Pain threshold [mN]	***p* = 0.033** (FDR: *p* = 0.131)	Δ = −1.18 (−2.39 to 0.04), *p* = 0.06	Δ = −0.03 (−1.32 to 1.25), *p* = 0.998	Δ = 1.14 (−0.07 to 2.36), *p* = 0.069
	Mechanical Pain sensitivity [mN]	*p* = 0.783	—	—	—
	Dynamic mechanical allodynia [no unit]	*p* = 0.534	—	—	—
	Wind‐up ratio [no unit]	*p* = 0.544	—	—	—
	Vibration detection threshold [no unit]	*p* = 0.152	—	—	—
	Pressure Pain Threshold, [kPa]	*p* = 0.184	—	—	—

*Note:* The mean and standard deviation values of the QST parameters for all groups are shown in original scale, but the group comparisons were performed using *z*‐scores, adjusted for age and sex, based on normative values. Note also that the pair‐wise group comparisons with Tukey post hoc test were performed only if the *F*‐test of ANOVA had a *p*‐value < 0.05 (thus only for: thermal sensory limen, mechanical detection threshold and mechanical pain threshold). Sensitivity analysis was performed excluding five patients with additional ipsilesional pain (CPSP 02, 03, 06, 14, 15). *p*‐values (< 0.05) are marked in bold.

Abbreviations: ∆, pairwise difference of the means; °C, degree Celsius; FDR, false discovery rate; kPa, kilopascal; mN, millinewton; QST, quantitative sensory testing.

Based on the results of the QST analysis, we designed a simple post hoc test to distinguish between thalamic CPSP and SCP by calculating a “mechanical index”. The index is based on the z‐scores of the mechanical detection and pain thresholds and is calculated by subtracting the mechanical pain threshold value from the mechanical detection threshold value, such that on average thalamic CPSP patients will have a high and SCP patients will have a low index value.

## 3 Results

### 3.1 Group Comparisons for Baseline Characteristics

There was no significant difference in age and sex among the three groups (thalamic CPSP, SCP and HC, Table 1).

The two thalamic stroke groups did not show significant differences at baseline regarding stroke laterality, severity and stroke type (ischemic vs. hemorrhagic) (Table 1). SCP had longer time since stroke compared to CPSP (Table 1), according to the inclusion criteria. Patients with CPSP had more sensory symptoms than SCP at the time of stroke and at study baseline (Table 1, Figures 3 and S1).

Most patients in both groups had ischemic strokes. The two most common causes of ischemic stroke were small‐vessel disease and cardioembolic stroke (Table 1). None of the recruited patients had suffered a previous ischemic, haemorrhagic, or traumatic brain injury other than the one leading to the study inclusion.

### 3.2 Pain Characteristics of CPSP Group

The pain characteristics of patients with CPSP are summarised in Table S1. In most patients, the onset of pain occurred relatively early after stroke, in 44% of patients within the first day after thalamic stroke and in 19% of patients within the first week and first month, respectively. Interestingly, two cases reported immediate onset of paresthesia and dysesthesia after stroke, with actual pain occurring 6–12 months later. The median pain duration at study baseline was 24 months (Table S1). The pain was described as permanent in 44%, followed by permanent with intermittent attacks in 31% of patients, and less frequently intermittent in 16% of patients. The average reported pain intensity on a numerical rating scale (NRS) was 5 out of 10 points (Table S1). In the CPSP group, 50% of patients were on symptomatic treatment (Table S1).

**FIGURE 1:**
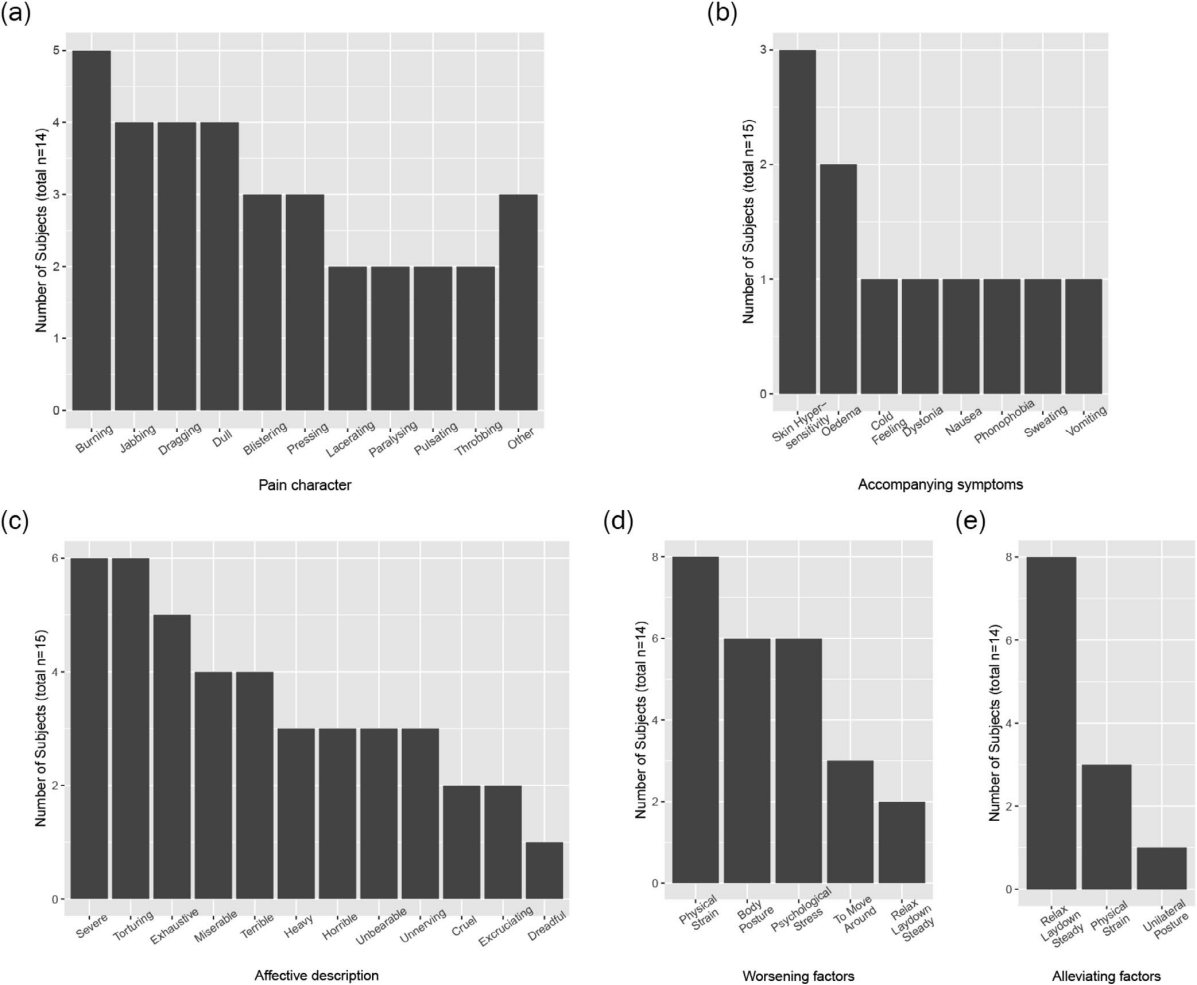
Pain characteristics in the CPSP group. The description of different pain characteristics is based on the patients' answers in the standardised pain questionnaire (“Deutscher Schmerzfragebogen”). (a) pain character, (b) accompanying symptoms (Skin hypersensitivity refers to the area of pain), (c) affective description, (d) worsening factors (Body posture refers to prolonged sitting/standing/walking) and (e) alleviating factors. Multiple answers were possible, *n* = number of participants who gave at least one answer (for some questions 1–2 missing).

Self‐reported pain characteristics (DGSS questionnaire) (Petzke et al. 2020) are summarised in Figure 1. The pain was most often described as burning (36% of patients), jabbing (29%), dragging (29%) or dull (29%), while the most common affective descriptions were severe (40% of patients), torturing (40%) and exhaustive (33%). Accompanying symptoms were rarely described; three patients reported hypersensitivity of the skin in the region affected by pain, and two subjects reported a feeling of swelling. The most common worsening factor was physical strain (57% of patients), followed by body posture (43%) and psychological stress (43%), while the most common alleviating factor was relaxing (57% of patients).

### 3.3 Lesion Localisation in the CPSP Group

The thalamic lesion location on MRI and lesion type in the CPSP group are summarised in Table S2. In all CPSP patients, the lesion was located in the lateral part of the thalamus, in the majority (11/16) more specifically in the posterior‐lateral part. Most patients had small, lacunar infarcts, while only three patients showed posterior infarctions with thalamus involvement and one a thalamic bleeding. Three characteristic MRI examples of lesion location are shown in Figure S2.

### 3.4 Group Comparisons of QST Parameters

#### 3.4.1 Temperature Thresholds

**FIGURE 2:**
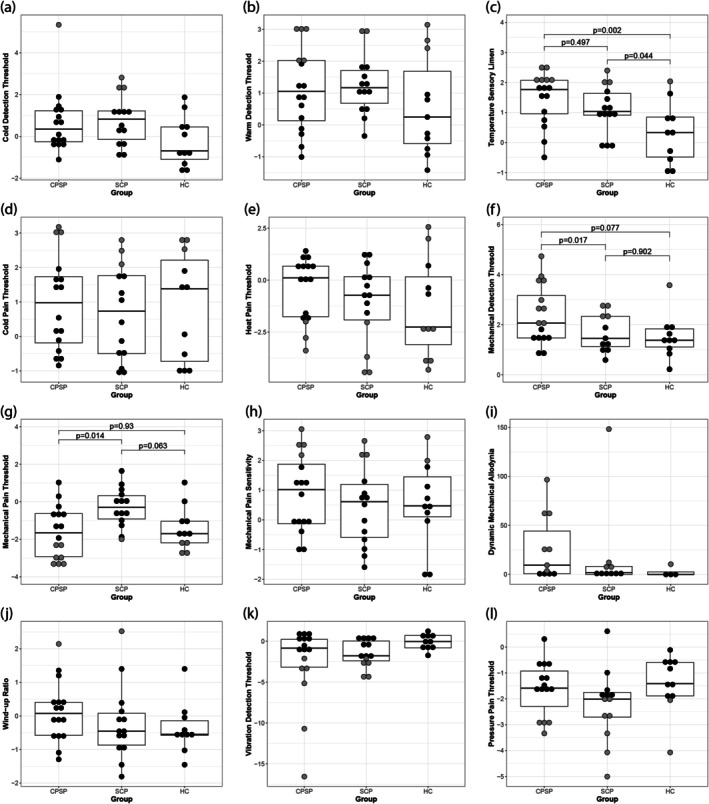
*Z*‐score values of all QST parameters per group. The age‐ and sex‐adjusted *Z*‐score values of all QST parameters (based on previously published reference data (Magerl et al. 2010)) are displayed in boxplots per study group. The following QST parameters are shown: (a) cold detection threshold (b) warm detection threshold (c) temperature sensory limen (d) cold pain threshold (e) heat pain threshold (f) mechanical detection threshold (g) mechanical pain threshold (h) mechanical pain sensitivity (i) dynamic mechanical allodynia (j) wind‐up‐ratio (k) vibration detection threshold (l) pressure pain threshold. Note that the *Z* score values which were outside the 95% CI are depicted in grey. The only parameters showing significant group‐differences were (c) temperature sensory limen, (f) mechanical detection‐ and (g) mechanical pain thresholds, the *p*‐values of these comparisons are shown in the figure.°C, Degree Celsius; CPSP, Central post stroke pain patients; HC, Healthy controls; mN, Millinewton; SCP, Stroke Control patients.

Both stroke groups (CPSP and SCP) had higher TSL (i.e., higher difference limen for alternating cold and warm stimuli) than HC on their contralesional hand (Figure 2, Table 2, Figure S3); but there was no difference between CPSP patients and SCP. The other temperature parameters did not show any group differences (Table 2).

#### 3.4.2 Mechanical and Pressure Thresholds

We detected significant group differences for mechanical detection thresholds (von Frey filaments) and mechanical pain thresholds (PinPrick stimulators) (Figure 2 Table 2). Patients with CPSP had significantly higher MDT on the contralesional hand than SCP (Figure 2, Table 3).

Moreover, patients with thalamic CPSP had significantly lower mechanical pain thresholds on the contralesional hand than SCP (Figure 2, Table 2), there was no difference between CPSP and HC. Thus, patients with CPSP were more sensitive to mechanical pain compared to patients with SCP.

Of note, the differences between CPSP and SCP for both the mechanical pain‐ and the mechanical detection thresholds remained significant after adjusting for time since stroke (Table 2).

#### 3.4.3 Other QST‐Parameters

We found no significant group differences for dynamic mechanical allodynia or for vibration detection thresholds (Table 2, Figures 2 and S3).

#### 3.4.4 Comparison of the Ipsilesional Hands

Group comparison of all QST parameters of the ipsilesional hand (not affected by stroke) revealed only higher MPT in CPSP vs. HC (CPSP‐HC: Δ = 1 (95% CI 0.07–1.9), *p* = 0.033), but no differences between CPSP and SCP.

In the within‐subject comparison (healthy vs. affected hand in CPSP and SCP) we found only a difference for the mechanical detection threshold in the CPSP group (mean difference affected minus healthy: 1.22 (95% CI 0.55–1.9), *p* = 0.002).

#### 3.4.5 Accuracy of Mechanical Detection and Mechanical Pain Thresholds for Distinguishing Patients With and Without CPSP

To combine the QST‐thresholds that showed the most pronounced differences between CPSP and SCP in a single variable that may be useful for differentiation between the groups, we devised a simple test combining the information from MDT and MPT: the “mechanical index” (see methods section on details). At a threshold of 2.87, sensitivity and specificity were balanced, and the “mechanical index” showed a specificity of 78.6% (true negative = 11, false positive = 3), sensitivity of 81.3% (true positive = 13, false negative = 3) and accuracy of 80% in distinguishing patients with CPSP from SCP (Figure S4).

### 3.5 Sensitivity Analyses

After excluding the five patients with additional ipsilesional pain, we found significantly higher MDT (CPSP vs. *SCP*: Δ = 1.43 (95% CI 0.29–2.56), *p* = 0.011) and, by trend, lower MPT in the CPSP vs. SCP (*p* = 0.06), supporting the findings of the main analysis. TSL was only different between CPSP and HC, as well as between SCP and HC, but not between CPSP and SCP (Table 3). Moreover, after excluding a single patient with an additional brain lesion (older stroke in the left frontoinsular cortex), the findings remained consistent with the main analysis (for MDT: CPSP vs. SCP Δ = 1.29 (95% CI 0.2–2.39), *p* = 0.017 and for MPT: CPSP vs. SCP Δ = 1.21 (95% CI −2.3 to −0.12), *p* = 0.027).

### 3.6 Analysis of Distribution Patterns of Pain and Other Sensory Symptoms in the CPSP Group

**FIGURE 3:**
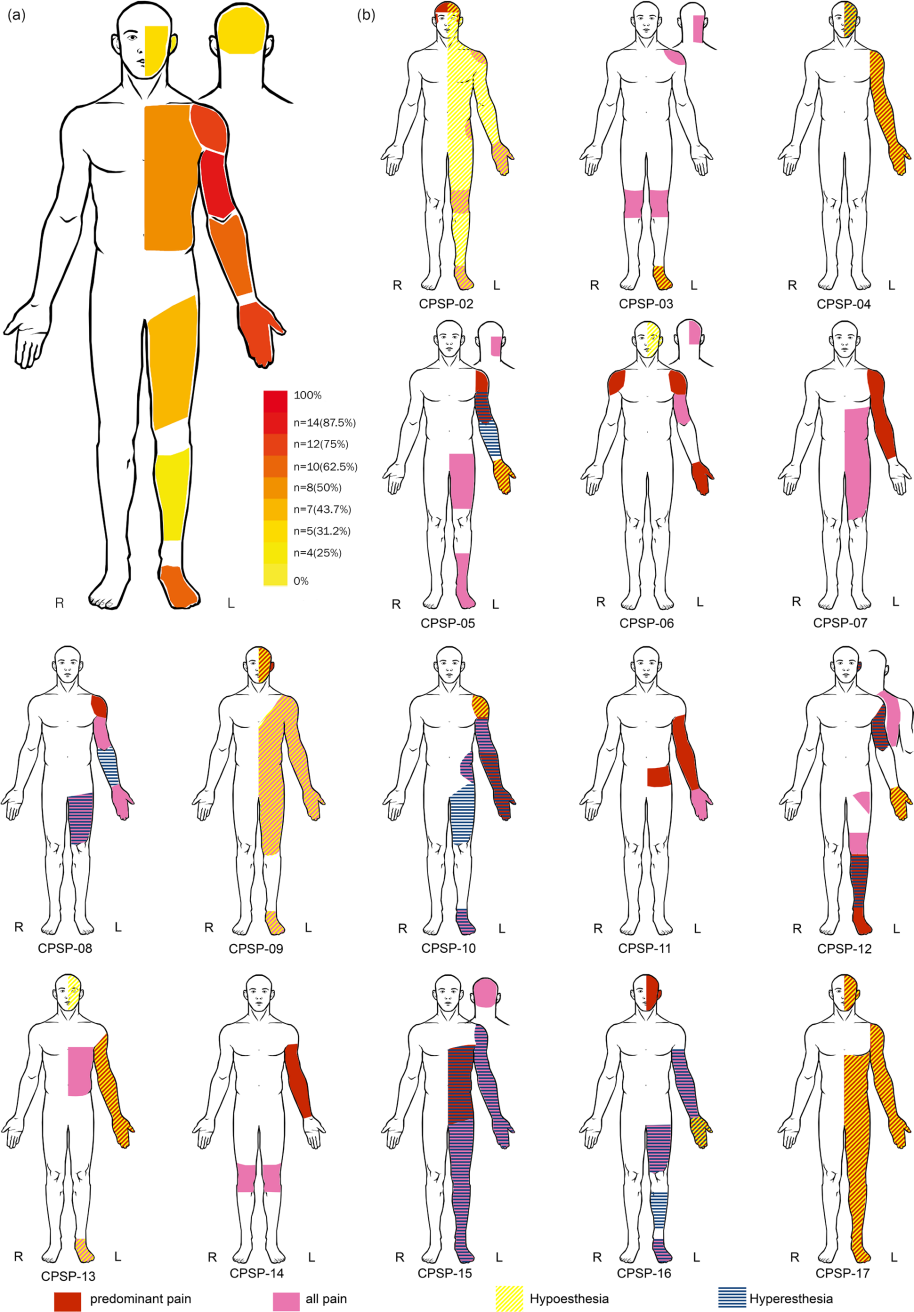
Topography of pain and heatmap of pain distribution in the CPSP group. (a) Visualisation of Heatmap showing the distribution of pain for specific body regions across all CPSP patients. Pain localised in the lower leg (*n* = 4), face, head (*n* = 5), upper leg (*n* = 7), trunk (*n* = 8), lower arm, foot (*n* = 10), hand, shoulder (*n* = 12) and upper arm (*n* = 14). Please note that the body areas affected by pain were assessed by a standardised pain questionnaire (“Deutscher Schmerzfragebogen”, DGSS, created by the German Pain Association, copyright: https://www.schmerzgesellschaft.de/). (b) Visualisation of Topography of pain and positive/negative sensory symptoms in the CPSP group “Predominant pain” (in red) refers to the body region with the most severe/frequent neuropathic pain. “All pain” (in pink) refers to any body region where the patients experienced pain, not related to the predominant neuropathic pain. “Hypesthesia” (yellow) refers to body regions that showed reduced sensitivity to light touch at bed‐side neurological examination, while “hyperesthesia” (blue bars) refers to positive symptoms revealed or reported by the patients at bed‐side examination (including paresthesia, dysesthesias, hyperesthesia for light touch and allodynia). All illustrations are standardised to show all symptoms on the left side.

The distribution of pain as well as other sensory symptoms (positive and negative, as defined above) in the CPSP group is depicted in Figure 3. Of the 16 patients suffering from CPSP, pain was localised on the upper arm in 87% and hand in 75%. Fewer patients (62.5%) had pain on the lower arm or foot (Figure 3a).

Finally, we analysed the overlap between the pain distribution and sensory signs and symptoms (Figure 3b). Regions with spontaneous pain coincided with negative sensory symptoms (hypoesthesia) in 35% of cases and positive sensory symptoms in 30% of cases. Vice versa, regions with hypoesthesia were painful in 77% and regions with positive sensory symptoms were painful in 90% of the cases. Note that in the CPSP group, the contralesional hand (which was also tested by QST) showed clinical sensory deficits in several patients (*n* = 7 with hypoesthesia, *n* = 2 with hyperesthesia and one with both hypo−/hyperesthesia). In the SCP group, seven patients had sensory deficits on the hand in bedside testing, with two involving both upper and lower extremities, including the hand (Figure S1).

## 4 Discussion

We quantified the somatosensory profile of patients with CPSP of thalamic origin, compared to patients with a history of thalamic stroke and no pain (SCP) as well as healthy controls.

We found that patients with CPSP had higher mechanical detection thresholds (i.e., hypoesthesia to light touch) compared to SCP; moreover, patients with CPSP showed lower mechanical pain detection thresholds compared to SCP.

These results suggest that a *short* QST protocol including mechanical testing could be useful in the diagnosis of CPSP.

Interestingly, we did not find significant group differences in temperature testing between CPSP and SCP. The temperature sensory limen was altered in both CPSP and SCP, but only compared to HC, indicating that this QST parameter was not as useful as mechanical thresholds for distinguishing between thalamic stroke with and without CPSP in our study.

A previous study (Vartiainen et al. 2016) including only thalamic CPSP and combining functional assessments (QST and laser‐evoked‐potentials) showed that mechanical parameters coexisted with thermal findings in more than half of the patients. Moreover, it was previously shown (Greenspan et al. 2004) that the presence of cold hypoesthesia is neither necessary nor sufficient to cause cold hyperalgesia in CPSP.

In a recent prospective study (Asseyer et al. 2025) mechanical detection threshold was significantly higher in patients with CPSP compared to stroke patients without pain, but only during the chronic phase and not acutely after stroke. In our study, patients were included at different time points after stroke, which could influence our results. However, a sensitivity analysis with adjustment for time since stroke did not show different results than the main analysis. Interestingly, the median time since stroke was rather long in our study (25 months). Thus, our results regarding mechanical thresholds align with those of Asseyer et al., and overall suggest that mechanical detection is impaired in patients with chronic CPSP.

Nevertheless, this recent prospective study also reported differences in thermal modalities, specifically cold‐ and warm detection thresholds, as well as heat pain thresholds, between patients with chronic CPSP and stroke controls. Prior studies (Barbosa et al. 2022; Boivie et al. 1989; Bowsher et al. 1998; (Bud) Craig 1998; Klit et al. 2009; Vestergaard et al. 1995) also suggested that alterations in temperature perception, particularly cold perception, are associated with the development of CPSP after thalamic stroke. In our study, CPSP and SCP did not differ in their temperature thresholds. However, thermal sensory limen was impaired in CPSP (and SCP) compared to healthy controls. Due to the examination of temperature perception only on the hand, we cannot exclude the role of thermal testing in diagnosing CPSP and differentiating it from thalamic stroke without pain. Moreover, the comparison of our findings with previous studies needs to be interpreted with caution due to methodological differences (e.g., studied body area, method of sensory testing etc.), the inclusion of partly different populations and the generally low number of patients studied due to the rarity of CPSP.

A previous study that also used QST (Krause, Asseyer, Geisler, et al. 2016) demonstrated differences mainly in temperature perception and DMA between CPSP and stroke controls on the affected body side. Interestingly, in that study, CPSP patients showed changes also in the ipsilesional body side. In our study, we found also an increased MPT on the non‐affected hand in CPSP compared to HC. These results support the involvement of bilateral cortical areas in the pathogenesis of thalamic CPSP (Krause, Asseyer, Taskin, et al. 2016). When looking at the distribution of pain and sensory findings (Figure 3), it has to be noted that the ipsilesional hand did not show abnormal findings. However, this was based on the bedside clinical testing, which is rather descriptive and not as sensitive as the QST parameters.

Earlier studies by (Klit et al. 2011, 2014) showed that early dysesthesia or allodynia at stroke onset was associated with a significantly increased risk of developing CPSP, while negative sensory findings (like hypoesthesia or anaesthesia) were not. In our study, we did not have accurate information regarding the exact sensory findings at the time of the stroke; however, median NIHSS (during the acute phase) was numerically higher in the CPSP vs. the SCP group. Moreover, onset of pain was reported by most patients early after stroke (in 88% within one month). Interestingly, two cases reported immediate onset of paresthesia and dysesthesia after the stroke, with pain symptoms appearing 6–12 months later, supporting the hypothesis of Klit et al. (2009) regarding early dysesthesia associated with the development of CPSP.

In this regard, we compared body regions with pain to body regions with positive (paresthesia, dysesthesia, hyperalgesia, allodynia) and negative sensory symptoms. We found that most (90%) of the regions with positive sensory symptoms were painful. However, many painful regions exhibited normal sensory perception, without positive or negative findings.

Although it has to be noted that these results derive from bedside testing, which might have limited sensitivity, the painful areas usually exceeded the areas with sensory deficits, and four patients reported bilateral pain. This supports the hypothesis that secondary plasticity mechanisms beyond the region directly damaged by stroke may be involved in the development of CPSP. This is also supported by a previous MRI study showing widespread, probably secondary cortical changes in patients with CPSP (Krause, Asseyer, Taskin, et al. 2016). Further evidence comes from a recent study (Asseyer et al. 2025) which also demonstrated ipsilesional QST abnormalities in the CPSP. To ensure that the presence of bilateral pain did not relevantly impact our results, we performed a sensitivity analysis excluding five patients reporting bilateral pain. All these patients reported the main pain on the contralateral side to the infarction but additional ipsilesional pain. This analysis confirmed our main findings.

Regarding allodynia, we did not find significantly higher DMA in CPSP vs. SCP; the bedside testing in our SCP group revealed overall mild sensory abnormalities (Figure S1) and no positive sensory symptoms like hyperalgesia or mechanical allodynia. The presence of allodynia in bedside testing could indeed be a helpful clinical sign in thalamic CPSP, as previously shown (Barbosa et al. 2022).

An important strength of our study is the comparison of CPSP to both HC/normative data and to “stroke control patients”, with no pain. This allowed us to detect changes merely attributed to stroke vs. changes specifically associated with the development of CPSP. Moreover, we used a quantified, standardised sensory testing method, while all subjects underwent detailed neurological interviews and examination and were diagnosed with CPSP by two experienced neurologists, applying strict, previously published criteria. This, together with the typical lesion location in the posterior‐lateral/lateral parts of the thalamus in the CPSP group, indicate that we included a typical group of “real‐world” patients with CPSP of thalamic origin.

The main limitation of our study is the cross‐sectional design, so that we could not assess the predictive value of our findings before the development of pain. Future longitudinal studies applying risk factors such as early dysesthesia (Klit et al. 2014) and a *short* QST protocol using mechanical testing to patients after thalamic stroke should confirm the value of our findings. Moreover, the QST was performed at different time points after the stroke/the onset of pain, which may represent a limitation and could have influenced our results. However, after adjustment of the analysis for time since stroke, the results remained consistent; thus we do not believe that this was a major drawback.

Another limitation is the small sample size of our study (due to the rarity of CPSP), which may not have allowed us to detect more subtle group differences (e.g., in thermal parameters). Due to the small sample size, we were unable to conduct more detailed phenotyping of the patients, for example, as proposed by a previous study (Vollert et al. 2017) and/or subgroup analyses according to the detailed pain profilin. We also did not include a group with thalamic stroke and other types of pain. Thus, the exact pathophysiological implications of our findings remain speculative. Still, our findings suggest that mechanical testing may be useful in patients with thalamic stroke, which is important from the clinical point of view, regardless of the exact underlying mechanisms.

Last, we analysed QST data only of the hand region and did not compare the findings of the clinical examination among the groups. This might be considered a limitation, since three of our CPSP patients did not have pain in the hand. However, two of these three patients still had pain in the upper extremity (a single patient had pain only in the foot), while the hand has the additional advantages of standardised normal values and less frequent affection by comorbidities like peripheral neuropathy (compared to the foot). In this regard, it has to be noted that four patients (*n* = 2 in CPSP and *n* = 2 in SCP) showed mild signs of peripheral neuropathy in bedside testing at study baseline. However, these were only located in the feet and were merely mild negative symptoms, without dysesthesia or pain. Thus, we do not expect a relevant impact on our findings through the inclusion of these patients.

To conclude, our results suggest that changes in mechanical thresholds are associated with CPSP of thalamic origin. A short somatosensory screening including mechanical perception (von Frey filaments, PinPricks) may contribute to the diagnosis of this distressing pain syndrome.

## Author Contributions

K.B. and L.E. perform literature research, acquisition of data, analysis and data interpretation, design the figures and tables and had a primary role in preparing the manuscript, which was finally approved by A.P. and T.S. A.P. and T.S. designed the study, developed the protocol, gained ethical approval, recruited patients, acquired funds, and interpreted data. A.P. performed the QST analysis. All authors reviewed and edited the manuscript and approved the final version of the manuscript.

## Conflicts of Interest

Kristel Berati, Priska Zuber and Kean Schoenhoelzer do not have conflicts of interest to declare. Lukas S. Enz has received funding from the Swiss National Science Foundation (323530_171139). Katarina Alexandra Ebner received compensation for advisory board (Lundbeck), which was used for research in the University Hospital of Basel. Federico Burguet Villena received travel support form TEVA. Laura Gaetano is currently an employee of Novartis AG. Ludwig Kappos: Institutional research support: steering committee, advisory board, consultancy fees: Actelion, Bayer HealthCare, Biogen, Bristol Myers Squibb, Genzyme, Janssen, Japan Tobacco, Merck, Novartis, Roche, Sanofi, Santhera, Shionogi, and TG Therapeutics, speaker fees: Bayer HealthCare, Biogen, Merck, Novartis, Roche, and Sanofi; support of educational activities: Allergan, Bayer HealthCare, Biogen, CSL Behring, Desitin, Genzyme, Merck, Novartis, Roche, Pfizer, Sanofi, Shire, and Teva; license fees for Neurostatus products; and grants: Bayer HealthCare, Biogen, European Union, Innosuisse, Merck, Novartis, Roche, Swiss MS Society, and Swiss National Research Foundation. Stefano Magon is currently an employee of F. Hoffmann‐La Roche. Athina Papadopoulou received speaker‐fees/fees for advisory boards/consulting activities from Sanofi‐Genzyme, Eli Lilly, AbbVie, Lundbeck and TEVA and travel support from Bayer AG, Teva and Hoffmann‐La Roche. Her research was supported by the University‐ and University Hospital of Basel, the Swiss Multiple Sclerosis Society, the “Stiftung zur Förderung der gastroenterologischen und allgemeinen klinischen Forschung sowie der medizinischen Bildauswertung”, the “Freie Akademische Gesellschaft Basel” and the Swiss National Science Foundation (Project number: P300PB_174480). During the current research work, Athina Papadopoulou was supported by the Swiss National Science Foundation (PZ00P3_216468). Till Sprenger received research grants from the Swiss MS Society, Novartis Pharmaceuticals Switzerland, EFIC‐Grünenthal grant, and Swiss National Science Foundation. The current (DKD Helios Klinik Wiesbaden) or previous (University Hospital Basel) institutions of Till Sprenger have received payments for speaking or consultation from: Biogen Idec, Eli Lilly, Allergan, Actelion, ATI, Mitsubishi Pharma, Novartis, Genzyme and TEVA.

## Supporting information


**Figure S1:** Sensory abnormalities in the SCP group. Visualisation of sensory abnormalities detected during physical examination at study visit in the stroke control patients. “Hypesthesia” (yellow) refers to body regions that showed reduced sensitivity to light touch at bed‐side neurological examination, while “hyperesthesia” (blue bars) refers to positive symptoms revealed or reported by the patients at bed‐side examination (including dysesthesias, hyperesthesia for light touch and allodynia). All illustrations are standardised to show all symptoms on the left side.

**Figure S2:** MRI Lesion localization in CPSP group. Magnetic resonance images in Fluid‐attenuated inversion recovery (FLAIR) image and diffusion‐weighted image (DWI) of three patients (CPSP05, CPSP10, CPSP 12). Lesion details for this patient are given in the Table S2.

**Figure S3:** Raw values of all QST parameters per group. The raw values of all measured QST parameters are displayed in boxplots per study group. (a) cold detection threshold (b) warm detection threshold (c) temperature sensory limen (d) cold pain threshold (e) heat pain threshold (f) mechanical detection threshold (g) mechanical pain threshold (h) mechanical pain sensitivity (i) dynamic mechanical allodynia (j) wind‐up‐ratio (k) vibration detection threshold (l) pressure pain threshold.°C, degree Celsius; CPSP, Central post stroke pain patients; HC, Healthy controls; mN, millinewton; SCP, Stroke Control patients.

**Figure S4:** Binary classification by mechanical detection‐ and mechanical pain thresholds. (a) Boxplots of index value calculated by subtracting the z‐transformed mechanical pain thresholds from the mechanical detection thresholds. Red dashed line shows optimal cut‐off value (2.87) at balanced sensitivity and specificity. (b) Sensitivity (black line) and specificity (red line) by cut‐off values. CPSP, Central post stroke pain patients; SCP, Stroke Control patients.

**Table S1:** Pain characteristics in the CPSP group. *These two patients had pain onset 6 months to 1 year after stroke, but immediate onset of par‐ and dysesthesias. **Several patients had tried other symptomatic treatments before, here only the current treatment at study‐baseline is listed.

**Table S2:** Lesion localisation in the CPSP Group. In 11/16 of the CPSP patient lesions were localised in the posterior‐lateral part of the thalamus, in five patients the lateral thalamus was involved. The majority, 12/16 patient had lacunar infarcts, only three patients showed posterior infarctions (with thalamus‐involvement) and just one had bleeding. *Magnetic resonance images of three patients (CPSP05, CPSP10, CPSP 12) are shown in Figure S2.

## References

[ejp70104-bib-0001] Andersen, G. , K. Vestergaard , M. Ingeman‐Nielsen , and T. S. Jensen . 1995. “Incidence of Central Post‐Stroke Pain.” Pain 61: 187–193. 10.1016/0304-3959(94)00144-4. 7659428

[ejp70104-bib-0002] Asseyer, S. , E. Panagoulas , J. Maidhof , et al. 2025. “Prediction of Central Post‐Stroke Pain by Quantitative Sensory Testing.” Annals of Neurology 97: 507–520. 10.1002/ana.27138. 39727056 PMC11831871

[ejp70104-bib-0003] Barbosa, L. M. , V. A. Da Silva , A. L. De Lima Rodrigues , et al. 2022. “Dissecting Central Post‐Stroke Pain: A Controlled Symptom‐Psychophysical Characterization.” Brain Communications 4: fcac090. 10.1093/braincomms/fcac090. 35528229 PMC9070496

[ejp70104-bib-0004] Bogousslavsky, J. , F. Regli , and A. Uske . 1988. “Thalamic Infarcts: Clinical Syndromes, Etiology, and Prognosis.” Neurology 38: 837. 10.1212/WNL.38.6.837. 3368064

[ejp70104-bib-0005] Boivie, J. , G. Leijon , and I. Johansson . 1989. “Central Post‐Stroke Pain—A Study of the Mechanisms Through Analyses of the Sensory Abnormalities.” Pain 37: 173–185. 10.1016/0304-3959(89)90128-0. 2748190

[ejp70104-bib-0007] Bowsher, D. 1996. “Central Pain: Clinical and Physiological Characteristics.” Journal of Neurology, Neurosurgery, and Psychiatry 61, no. 1: 62–69. 10.1136/jnnp.61.1.62. 8676164 PMC486461

[ejp70104-bib-0006] Bowsher, D. , G. Leijon , and K.‐A. Thuomas . 1998. “Central Poststroke Pain: Correlation of MRI With Clinical Pain Characteristics and Sensory Abnormalities.” Neurology 51: 1352–1358. 10.1212/WNL.51.5.1352. 9818859

[ejp70104-bib-0008] Craig, A. D. (B.) . 1998. “A New Version of the Thalamic Disinhibition Hypothesis of Central Pain.” Pain Forum 7: 1–14. 10.1016/S1082-3174(98)70004-2.

[ejp70104-bib-0009] Déjerine, J. , and G. Roussy . 1906. “Le Syndrome Thalamique.” Revue Neurologique 14: 521–532.

[ejp70104-bib-0010] Delboni Lemos, M. , I. Faillenot , L. Tavares Lucato , et al. 2022. “Dissecting Neuropathic From Poststroke Pain: The White Matter Within.” Pain 163: 765–778. 10.1097/j.pain.0000000000002427. 35302975

[ejp70104-bib-0011] Garcia‐Larrea, L. , C. Perchet , C. Creac'h , et al. 2010. “Operculo‐Insular Pain (Parasylvian Pain): A Distinct Central Pain Syndrome.” Brain 133: 2528–2539. 10.1093/brain/awq220. 20724291

[ejp70104-bib-0012] Greenspan, D. J. , S. Ohara , E. Sarlani , and A. F. Lenz . 2004. “Allodynia in Patients With Post‐Stroke Central Pain (CPSP) Studied by Statistical Quantitative Sensory Testing Within Individuals.” Pain 109: 357–366. 10.1016/j.pain.2004.02.002. 15157697

[ejp70104-bib-0013] Harno, H. , E. Haapaniemi , J. Putaala , et al. 2014. “Central Poststroke Pain in Young Ischemic Stroke Survivors in the Helsinki Young Stroke Registry.” Neurology 83: 1147–1154. 10.1212/WNL.0000000000000818. 25128182

[ejp70104-bib-0014] Henry, J. L. , C. Lalloo , and K. Yashpal . 2008. “Central Poststroke Pain: An Abstruse Outcome.” Pain Research & Management 13: 41–49. 10.1155/2008/754260. 18301815 PMC2670809

[ejp70104-bib-0015] Klit, H. , N. B. Finnerup , G. Andersen , and T. S. Jensen . 2011. “Central Poststroke Pain: A Population‐Based Study.” Pain 152: 818–824. 10.1016/j.pain.2010.12.030. 21272999

[ejp70104-bib-0016] Klit, H. , N. B. Finnerup , and T. S. Jensen . 2009. “Central Post‐Stroke Pain: Clinical Characteristics, Pathophysiology, and Management.” Lancet Neurology 8: 857–868. 10.1016/S1474-4422(09)70176-0. 19679277

[ejp70104-bib-0017] Klit, H. , A. P. Hansen , N. S. Marcussen , N. B. Finnerup , and T. S. Jensen . 2014. “Early Evoked Pain or Dysesthesia Is a Predictor of Central Poststroke Pain.” Pain 155: 2699–2706. 10.1016/j.pain.2014.09.037. 25284071

[ejp70104-bib-0018] Kong, K.‐H. , V.‐C. Woon , and S.‐Y. Yang . 2004. “Prevalence of Chronic Pain and Its Impact on Health‐Related Quality of Life in Stroke Survivors.” Archives of Physical Medicine and Rehabilitation 85: 35–40. 10.1016/S0003-9993(03)00369-1. 14970965

[ejp70104-bib-0019] Krause, T. , S. Asseyer , F. Geisler , et al. 2016. “Chronic Sensory Stroke With and Without Central Pain Is Associated With Bilaterally Distributed Sensory Abnormalities as Detected by Quantitative Sensory Testing.” Pain 157: 194–202. 10.1097/j.pain.0000000000000354. 26397931

[ejp70104-bib-0020] Krause, T. , S. Asseyer , B. Taskin , et al. 2016. “The Cortical Signature of Central Poststroke Pain: Gray Matter Decreases in Somatosensory, Insular, and Prefrontal Cortices.” Cerebral Cortex 26: 80–88. 10.1093/cercor/bhu177. 25129889

[ejp70104-bib-0021] Krause, T. , P. Brunecker , S. Pittl , et al. 2012. “Thalamic Sensory Strokes With and Without Pain: Differences in Lesion Patterns in the Ventral Posterior Thalamus.” Journal of Neurology, Neurosurgery, and Psychiatry 83: 776–784. 10.1136/jnnp-2011-301936. 22696587

[ejp70104-bib-0022] Lampl, C. , K. Yazdi , and C. Röper . 2002. “Amitriptyline in the Prophylaxis of Central Poststroke Pain: Preliminary Results of 39 Patients in a Placebo‐Controlled, Long‐Term Study.” Stroke 33: 3030–3032. 10.1161/01.STR.0000037674.95228.86. 12468808

[ejp70104-bib-0023] Leijon, G. , J. Boivie , and I. Johansson . 1989. “Central Post‐Stroke Pain—Neurological Symptoms and Pain Characteristics.” Pain 36: 13–25. 10.1161/01.STR.0000037674.95228.86. 2919091

[ejp70104-bib-0024] Loeser, J. D. , and R.‐D. Treede . 2008. “The Kyoto Protocol of IASP Basic Pain Terminology.” Pain 137: 473–477. 10.1016/0304-3959(89)90107-3. 18583048

[ejp70104-bib-0025] Magerl, W. , E. K. Krumova , R. Baron , T. Tölle , R.‐D. Treede , and C. Maier . 2010. “Reference Data for Quantitative Sensory Testing (QST): Refined Stratification for Age and a Novel Method for Statistical Comparison of Group Data.” Pain 151: 598–605. 10.1016/j.pain.2010.07.026. 20965658

[ejp70104-bib-0026] Paciaroni, M. , and J. Bogousslavsky . 1998. “Pure Sensory Syndromes in Thalamic Stroke.” European Neurology 39: 211–217. 10.1016/j.jpain.2008.06.013. 9635471

[ejp70104-bib-0027] Petzke, F. , M. Hüppe , T. Kohlmann , et al. 2020. Handbuch Deutscher Schmerz‐Fragebogen .

[ejp70104-bib-0028] R Development Core Team . 2010. R: A Language and Environment for Statistical Computing. R Foundation for Statistical Computing.

[ejp70104-bib-0029] Rolke, R. , W. Magerl , K. A. Campbell , et al. 2006. “Quantitative Sensory Testing: A Comprehensive Protocol for Clinical Trials.” European Journal of Pain 10: 77. 10.1016/j.ejpain.2005.02.003. 16291301

[ejp70104-bib-0030] Sprenger, T. , C. L. Seifert , M. Valet , et al. 2012. “Assessing the Risk of Central Post‐Stroke Pain of Thalamic Origin by Lesion Mapping.” Brain 135: 2536–2545. 10.1093/brain/aws153. 22719000

[ejp70104-bib-0031] Treede, R.‐D. , T. S. Jensen , J. N. Campbell , et al. 2008. “Neuropathic Pain: Redefinition and a Grading System for Clinical and Research Purposes.” Neurology 70: 1630–1635. 10.1212/01.wnl.0000282763.29778.59. 18003941

[ejp70104-bib-0032] Truelsen, T. , B. Piechowski‐Jóźwiak , R. Bonita , C. Mathers , J. Bogousslavsky , and G. Boysen . 2006. “Stroke Incidence and Prevalence in Europe: A Review of Available Data.” European Journal of Neurology 13: 581–598. 10.1111/j.1468-1331.2006.01138.x. 16796582

[ejp70104-bib-0033] Vartiainen, N. , C. Perchet , M. Magnin , et al. 2016. “Thalamic Pain: Anatomical and Physiological Indices of Prediction.” Brain 139: 708–722. 10.1093/brain/awv389. 26912644

[ejp70104-bib-0034] Vestergaard, K. , J. Nielsen , G. Andersen , M. Ingeman‐Nielsen , L. Arendt‐Nielsen , and T. S. Jensen . 1995. “Sensory Abnormalities in Consecutive, Unselected Patients With Central Post‐Stroke Pain.” Pain 61: 177–186. 10.1016/0304-3959(94)00140-A. 7659427

[ejp70104-bib-0035] Vollert, J. , C. Maier , N. Attal , et al. 2017. “Stratifying Patients With Peripheral Neuropathic Pain Based on Sensory Profiles: Algorithm and Sample Size Recommendations.” Pain 158: 1446–1455. 10.1097/j.pain.0000000000000935. 28595241 PMC5515640

[ejp70104-bib-0036] Widar, M. , L. Samuelsson , S. Karlsson‐Tivenius , and G. Ahlström . 2002. “Long‐Term Pain Conditions After a Stroke.” Journal of Rehabilitation Medicine 34: 165–170. 10.1080/16501970213237. 12201611

