# Supplementary figures and images for: Somatosensory Profile of Central Post Stroke Pain of Thalamic Origin: Findings of a Quantitative Sensory Testing Study
Source: Eur J Pain. 2025 Aug 15;29(8):e70104. doi: 10.1002/ejp.70104 (PMC12355633; doi:10.1002/ejp.70104)

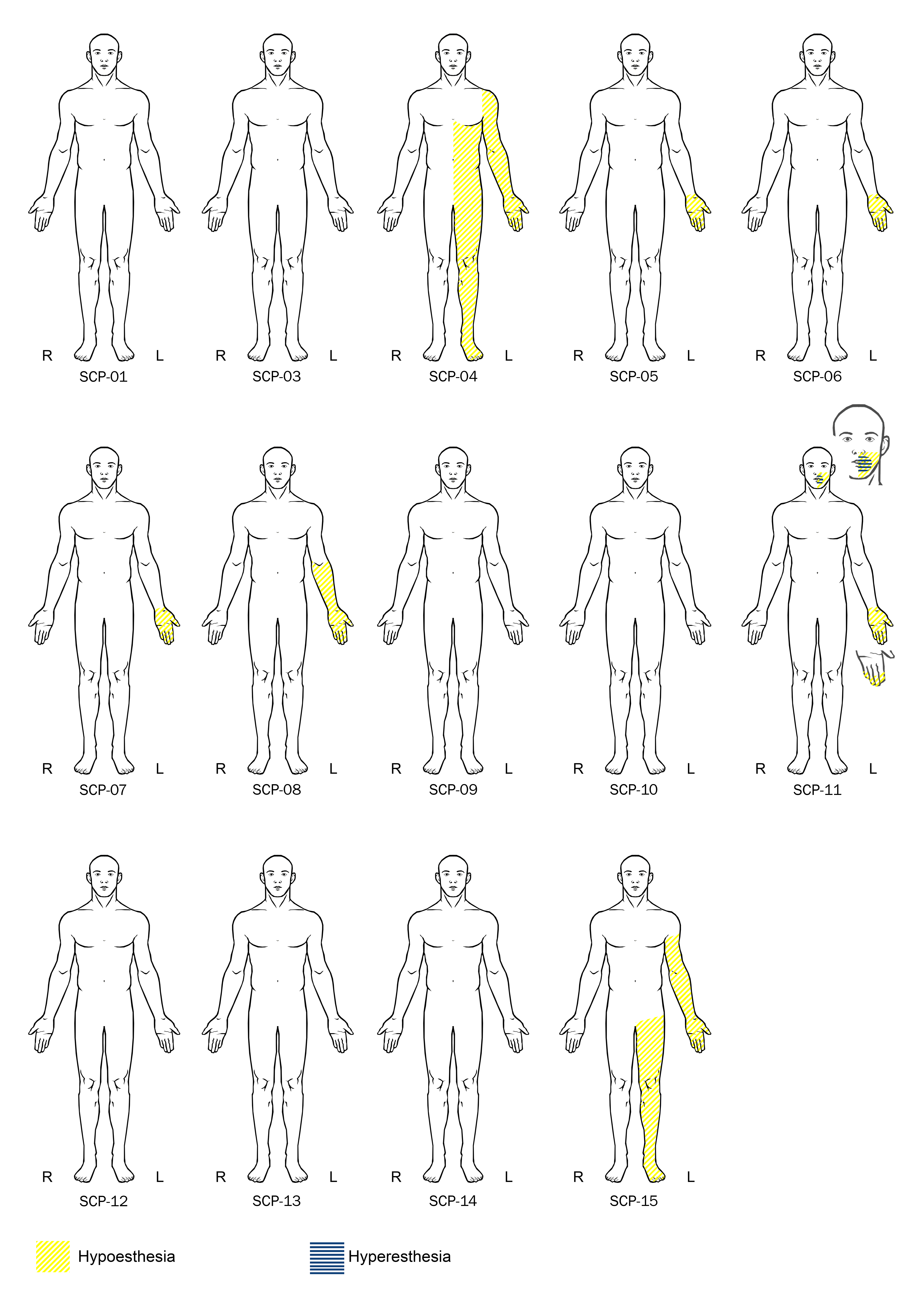

Supplement: Supplementary file 1 — Figure S1: Sensory abnormalities in the SCP group. Visualisation of sensory abnormalities detected during physical examination at study visit in the stroke control patients. “Hypesthesia” (yellow) refers to body regions that showed reduced sensitivity to light touch at bed‐side neurological examination, while “hyperesthesia” (blue bars) refers to positive symptoms revealed or reported by the patients at bed‐side examination (including dysesthesias, hyperesthesia for light touch and allodynia). All illustrations are standardised to show all symptoms on the left side. [file EJP-29-0-s001.png]

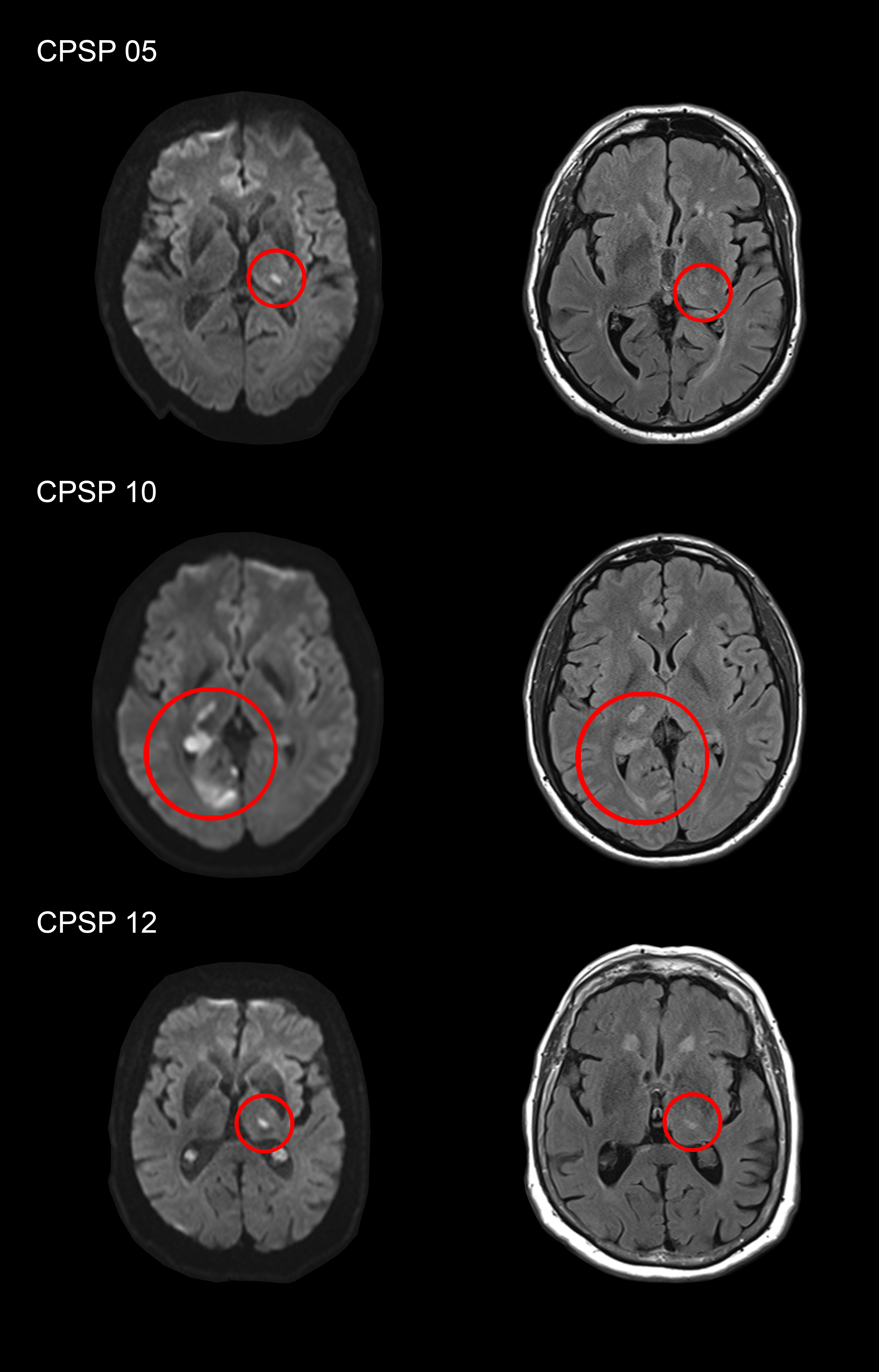

Supplement: Supplementary file 2 — Figure S2: MRI Lesion localization in CPSP group. Magnetic resonance images in Fluid‐attenuated inversion recovery (FLAIR) image and diffusion‐weighted image (DWI) of three patients (CPSP05, CPSP10, CPSP 12). Lesion details for this patient are given in the Table S2. [file EJP-29-0-s006.png]

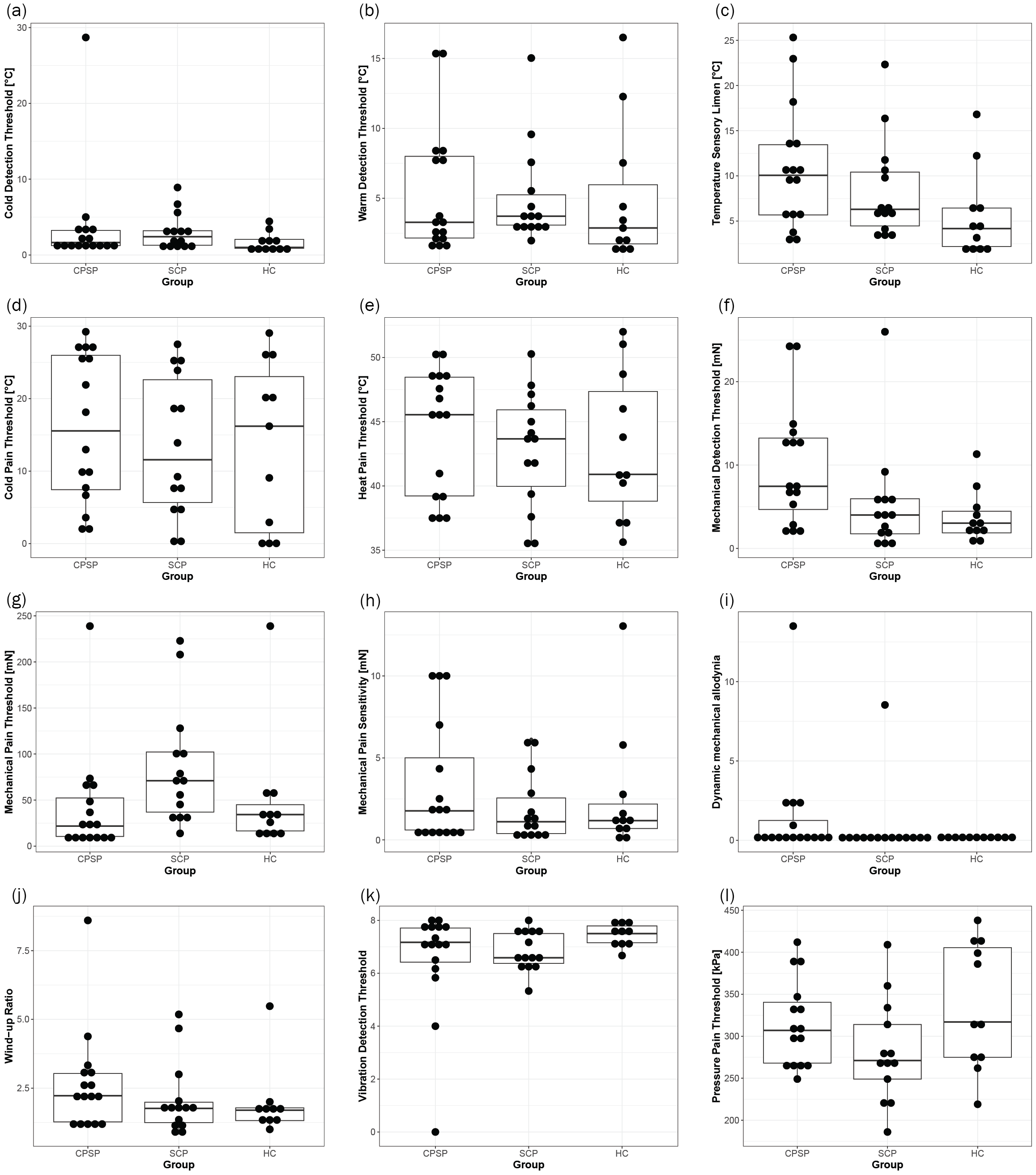

Supplement: Supplementary file 3 — Figure S3: Raw values of all QST parameters per group. The raw values of all measured QST parameters are displayed in boxplots per study group. (a) cold detection threshold (b) warm detection threshold (c) temperature sensory limen (d) cold pain threshold (e) heat pain threshold (f) mechanical detection threshold (g) mechanical pain threshold (h) mechanical pain sensitivity (i) dynamic mechanical allodynia (j) wind‐up‐ratio (k) vibration detection threshold (l) pressure pain threshold.°C, degree Celsius; CPSP, Central post stroke pain patients; HC, Healthy controls; mN, millinewton; SCP, Stroke Control patients. [file EJP-29-0-s004.png]

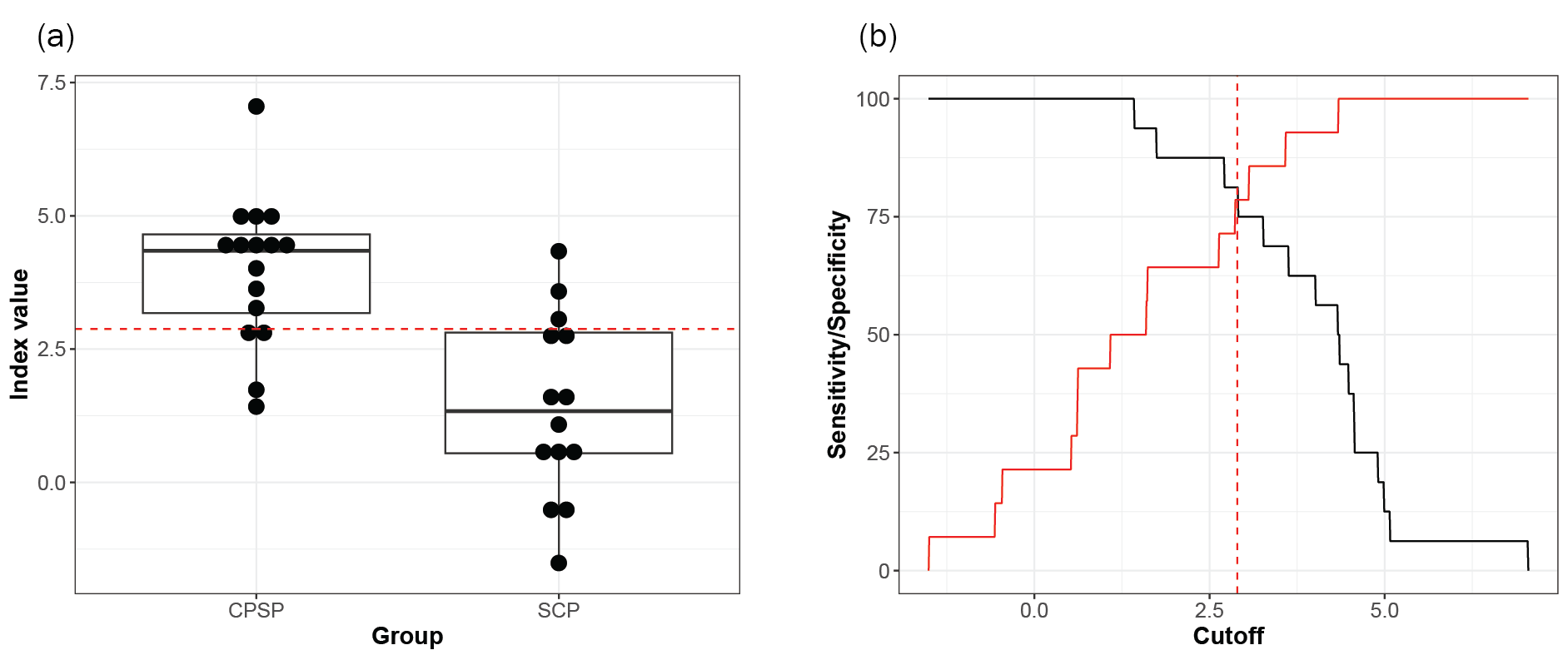

Supplement: Supplementary file 4 — Figure S4: Binary classification by mechanical detection‐ and mechanical pain thresholds. (a) Boxplots of index value calculated by subtracting the z‐transformed mechanical pain thresholds from the mechanical detection thresholds. Red dashed line shows optimal cut‐off value (2.87) at balanced sensitivity and specificity. (b) Sensitivity (black line) and specificity (red line) by cut‐off values. CPSP, Central post stroke pain patients; SCP, Stroke Control patients. [file EJP-29-0-s003.png]
