# Supplementary material for: Somatosensory Profile of Central Post Stroke Pain of Thalamic Origin: Findings of a Quantitative Sensory Testing Study
Source: Eur J Pain. 2025 Aug 15;29(8):e70104. doi: 10.1002/ejp.70104 (PMC12355633; doi:10.1002/ejp.70104)
Supplement: Supplementary file 5 — Table S1: Pain characteristics in the CPSP group. *These two patients had pain onset 6 months to 1 year after stroke, but immediate onset of par‐ and dysesthesias. **Several patients had tried other symptomatic treatments before, here only the current treatment at study‐baseline is listed. [file EJP-29-0-s002.docx]

**Supplementary Table 1: Pain characteristics in the CPSP-group**

| **Characteristics** |  |
| --- | --- |
| Onset of Pain after Stroke | N (%) |
| Within the 1^st^ day | 7 (44%) |
| Within 1 week | +3 (19%) |
| Within 1 month | +3 (19%) |
| Up to 3 months | +1 (6%) |
| 6 months up to 1 year | +2* (12%) |
| Pain duration in months: median (range: min-max) | 24 (1- 249) |
| Pain pattern: N (%) |  |
| Permanent | 7 (44%) |
| Permanent with intermittent attacks | 5 (31%) |
| Intermittent | 3 (19%) |
| Unknown | 1 (6%) |
| Average pain intensity in the last 4 weeks (NRS^[[1]](#footnote-1)^ 0-10)  Median (range) | 5 (1-9) |
| Minimum pain intensity in the last 4 weeks  Median (range) | 2 (1-5) |
| Maximum pain intensity in the last 4 weeks  Median (range) | 7.5 (1-10) |
| Symptomatic treatment for pain at baseline N (%) | 8 (50%) ** |
| Paracetamol/Acetaminophen | 1 (6.25%) |
| Tramadol | 1 (6.25%) |
| Gabapentin | 2 (12.5%) |
| Pregabalin | 2 (12.5%) |
| Trimipramine | 1 (6.25%) |
| Carbamazepine | 1 (6.25%) |
| Venlafaxine | 1 (6.25%) |

**Legend Supplementary Table 1:** *These two patients had pain onset 6 months to 1 year after stroke, but immediate onset of par- and dysesthesias. ** Several patients had tried other symptomatic treatments before, here only the current treatment at study-baseline is listed.

1. NRS= numerical rating scale [↑](#footnote-ref-1)
