# Supplementary material for: Somatosensory Profile of Central Post Stroke Pain of Thalamic Origin: Findings of a Quantitative Sensory Testing Study
Source: Eur J Pain. 2025 Aug 15;29(8):e70104. doi: 10.1002/ejp.70104 (PMC12355633; doi:10.1002/ejp.70104)
Supplement: Supplementary file 6 — Table S2: Lesion localisation in the CPSP Group. In 11/16 of the CPSP patient lesions were localised in the posterior‐lateral part of the thalamus, in five patients the lateral thalamus was involved. The majority, 12/16 patient had lacunar infarcts, only three patients showed posterior infarctions (with thalamus‐involvement) and just one had bleeding. *Magnetic resonance images of three patients (CPSP05, CPSP10, CPSP 12) are shown in Figure S2. [file EJP-29-0-s005.docx]

| **Patient-code** | **Localization** | | **Lesiontype** | **Shown in figure** |
| --- | --- | --- | --- | --- |
|  | **Side** | **Part of Thalamus** |  |  |
| CPSP02 | Right | Lateral | Posterior Infarction |  |
| CPSP03 | Left | Posterior-lateral | Lacune |  |
| CPSP04 | Right | Lateral | Lacune |  |
| CPSP05 | Left | Posterior-lateral | Lacune | * |
| CPSP06 | Right | Lateral | Lacune |  |
| CPSP07 | Right | Posterior-lateral | Lacune |  |
| CPSP08 | Left | Posterior-lateral | Posterior Infarction |  |
| CPSP09 | Left | Posterior-lateral | Bleeding |  |
| CPSP10 | Right | Posterior-lateral | Posterior Infarction | * |
| CPSP11 | Left | Posterior-lateral | Lacune |  |
| CPSP12 | Left | Posterior-lateral | Lacune | * |
| CPSP13 | Right | Posterior-lateral | Lacune |  |
| CPSP14 | Left | Lateral | Lacune |  |
| CPSP15 | Right | Posterior-lateral | Lacune |  |
| CPSP16 | Left | Lateral | Lacune |  |
| CPSP17 | Right | Posterior-lateral | Lacune |  |
| *Summary CPSP* | *8 Right*  *8 Left* | *5 Lateral*  *11 Posterior-lateral* | *3 Posterior Infarction*  *12 Lacune*  *1 Bleeding* |  |

**Supplementary Table 2: Lesion localization and type in the CPSP group**

**Legend Supplementary Table 2**: *In 11/16 of the CPSP patient lesions were localized in the posterior-lateral part of the thalamus, were in five patients the lateral thalamus was involved. The majority, 12/16 patient had lacunar infarcts, only three patients showed posterior infarctions (with thalamus-involvement) and just one had bleeding.*

** Magnetic resonance images of three patients (CPSP05, CPSP10, CPSP 12) are shown in supplementary figure 2..*
